# Falls in a single brain rehabilitation center: a 3-year retrospective chart review

**DOI:** 10.3389/fneur.2025.1519555

**Published:** 2025-02-24

**Authors:** Yoo Jin Choo, Jun Sung Moon, Gun Woo Lee, Wook-Tae Park, Min Cheol Chang

**Affiliations:** ^1^Department of Physical Medicine and Rehabilitation, College of Medicine, Yeungnam University, Daegu, Republic of Korea; ^2^Division of Endocrinology and Metabolism, Department of Internal Medicine, College of Medicine, Yeungnam University, Daegu, Republic of Korea; ^3^Department of Orthopaedic Surgery, College of Medicine, Yeungnam University, Daegu, Republic of Korea

**Keywords:** fall, brain lesion, stroke, brain tumor, risk factor

## Abstract

**Objective:**

Falls in brain rehabilitation centers can negatively impact patient recovery, increase injury risk, and adversely affect rehabilitation outcomes. This study aimed to analyze the incidence of falls and identify associated risk factors among patients with brain lesions in a tertiary hospital’s brain rehabilitation center from June 2021 to May 2024.

**Methods:**

A retrospective chart review was conducted to examine patient characteristics, fall-related risk factors, functional assessments, and circumstances surrounding falls.

**Results:**

Among 316 patients, 10 (3.2%) experienced falls, with a mean age of 61.3 ± 11.9 years. Most patients demonstrated walking and cognitive impairments. Seven falls (70%) occurred during attempts to get out of bed, with five of these incidents occurring at night. Falls were observed despite the presence of caregivers and bed rails. Additionally, all 10 patients were administered medications that may increase fall risk, such as somnifacients or tranquilizers. The occurrence of falls appeared to be associated with multiple factors, including physical limitations (e.g., impaired mobility, visual disturbances, and medication side effects), environmental conditions (e.g., inadequate lighting and medical staff shortages), and behavioral aspects (e.g., unassisted movement).

**Conclusion:**

To mitigate fall risk, a comprehensive approach that includes improved nighttime lighting, education on assistive device use and medication management, caregiver training, and personalized rehabilitation programs may be beneficial.

## 1 Introduction

Brain rehabilitation centers serve as critical medical institutions for the recovery and rehabilitation of patients with brain injuries. These centers function as an essential intermediary stage to help patients in their transition to daily life by facilitating the recovery of both physical and cognitive functions, thereby enabling them to achieve the highest possible level of independence (1). In particular, brain rehabilitation requires a comprehensive approach that involves not only physical recovery but also cognitive recovery, sensory integration, and emotional well-being (2). However, patients with brain injuries face numerous challenges throughout the rehabilitation process; one such challenge is the risk of falls (3).

Falls occurring within rehabilitation centers are a significant issue because they can negatively impact the safety and recovery of patients. Patients with brain injuries are at an elevated risk of falls because of various factors, such as physical weakness, impaired balance, and medication use (4). This risk is particularly pronounced during rehabilitation, when patients may not have fully regained their physical abilities (5). Furthermore, a combination of cognitive impairments and motor dysfunction resulting from acquired brain injury increases the risk of falls. The incidence of falls in inpatient rehabilitation facilities has been reported to be as high as 15.9 per 1,000 days, whereas in general hospitals, the maximum rate is 3.73 per 1,000 days, thereby indicating a substantially higher fall rate in rehabilitation wards (6). Furthermore, a study by Callaly et al. (7) reported that approximately 24% of stroke patients experienced at least one fall within 2 years. Additionally, Teasell et al. (8) documented 180 fall incidents among 88 (37%) of 238 patients hospitalized in a rehabilitation ward over a 5-year period. Among these 88 patients, 45 experienced a single fall, 25 fell twice, 9 fell three times, and another 9 experienced four or more falls, thereby underscoring the prevalence of recurrent falls. Similarly, Lee et al. (9) reported that 34 (20.48%) of 166 patients hospitalized in a rehabilitation ward over the course of 1 year experienced falls. Falls within rehabilitation centers are more than mere accidents; they can lead to serious complications, including physical injury, delayed recovery, increased treatment costs, and worsened rehabilitation outcomes (10). These issues hinder the recovery process and may have detrimental effects on the psychological well-being of patients.

Therefore, fall prevention in rehabilitation centers should be addressed as a crucial matter requiring customized approaches that consider the unique characteristics and conditions of each patient (11). It is essential to perform comprehensive evaluations that encompass various factors, including physical weakness, psychological state, and emotional state, and develop appropriate preventive strategies accordingly (12). Such personalized approaches can reduce the risk of falls in individual patients and contribute to more successful rehabilitation outcomes.

The objective of this study was to propose practical solutions to reduce fall rates in rehabilitation centers and enhance patient recovery and rehabilitation outcomes. To this end, we conducted a 3-year analysis of fall incidence and associated risk factors among patients admitted to a rehabilitation center. Furthermore, we identified the underlying causes of falls and discussed strategies for mitigating fall risks.

## 2 Methods

A retrospective database review was conducted for patients admitted to the brain rehabilitation center at Yeungnam University Hospital over 3 years, from June 2021 to May 2024. The registered patients were hospitalized for brain rehabilitation due to stroke, traumatic brain injury, or brain tumors. There were no specific inclusion or exclusion criteria, and all cases of falls occurring during hospitalization were included in the analysis. This study was approved by the Institutional Review Board of Yeungnam University Hospital, and written informed consent was waived due to the retrospective nature of the study. This study conforms to all STROBE guidelines and reports the required information accordingly.

### 2.1 Patient characteristics and clinical assessment

Data regarding the general characteristics of the patients were collected. The collected data included age, sex, medical history, medical condition at the time of the fall, fall risk status, type of rehabilitation therapy performed at the time of the fall, gait and cognitive function assessed on the day of or the day before the fall, and use of medications that might increase fall risk (e.g., somnifacients, tranquilizers, or agents for benign prostatic hyperplasia). Individuals with a Morse Fall Scale score of 51 or higher were considered to have a high risk of falls (13). The Morse Fall Scale is a tool used to assess fall risk by evaluating various factors, such as the patient’s fall history, walking status, and mental status (13). Typically, a score below 25 indicates low risk, a score ranging from 25 to 50 indicates moderate risk, and a score of 51 or higher indicates high risk. Gait function was assessed using the Functional Ambulation Categories (FAC) (14), and cognitive function was evaluated using the Mini-Mental State Examination (MMSE) (15). The FAC is a tool for assessing independent walking ability; the FAC score is categorized into six levels: 0, unable to walk independently and requires the assistance of two or more people; 1, requires continuous and significant assistance from one person to maintain weight shift and balance; 2, requires intermittent assistance from one person to maintain balance; 3, requires verbal correction without physical contact; 4, capable of walking independently indoors but requires assistance on stairs or uneven surfaces; and 5, capable of walking independently both indoors and outdoors. The MMSE is a tool for assessing cognitive function after acquired brain injury; it consists of 11 domains. An MMSE score of 24 or higher is considered normal, a score from 20 to 23 indicates mild cognitive impairment, and a score below 20 indicates moderate to severe cognitive impairment.

### 2.2 Definition and classification of falls

In this study, a fall was defined as an incident in which a patient descends to the floor from a sitting or standing position, unexpectedly falls to the floor while walking, or descends from a bed and falls to the ground. Falls were identified through self-reports by patients, reports from caregivers, or observations by medical staff. When a fall occurred, the medical staff documented the circumstances and the patient’s condition at the time of the fall in the electronic medical record. The fall-related data collected in this study included the time of the fall, presence of a caregiver during the fall, location of the fall, presence of bed rails, patient’s level of consciousness during the fall, and resulting injuries. The time of the fall was categorized into daytime and nighttime (9:30 p.m. to 6:30 a.m.). The location was classified as occurring in bed, during ambulation, or while using a wheelchair. The level of consciousness during the fall was categorized as alert, drowsy, stuporous, confused, or comatose. If any patient reported pain, indicated that they had struck a part of their body, or exhibited a visible abnormality, imaging studies, such as computed tomography scans or X-rays, were recommended. Imaging was performed with the consent of the patient or caregiver; if they declined, it was not performed.

### 2.3 Injury classification

The information regarding injuries, as documented in the medical records, was descriptive in nature and did not follow a standardized format. Based on previous studies (16), the severity of injuries was classified as follows: (1) no injury; (2) mild injury: minor cuts, minor bleeding, skin abrasions, swelling, pain, and minor contusions; (3) moderate injury: excessive bleeding, lacerations requiring sutures, temporary loss of consciousness, and moderate head trauma; and (4) severe injury: fractures, subdural hematomas, other major head trauma, cardiac arrest, and death.

### 2.4 Statistical analysis

The patients’ age, FAC scores, and MMSE scores were described using means and standard deviations, and the data were analyzed using jamovi software, version 2.3.

## 3 Results

Of the 316 patients admitted to the brain rehabilitation center over the past 3 years, 10 patients (3.2%) experienced falls during hospitalization. The average age of these 10 patients was 61.3 ± 11.9 years (range: 39 to 82 years); six patients were male (60%) and four were female (40%). Nine patients (90%) had been diagnosed as having a stroke, while one patient (10%) had a brain tumor. Four patients (40%) had hemiplegia, and six patients (60%) had quadriplegia. Three patients (30%) had a history of hypertension, and three patients (30%) had a history of diabetes. All patients were undergoing both physical and occupational therapies. The mean FAC score was 2.4 ± 1.1 (range: 1–4), indicating significant impairment in independent walking. The mean MMSE score was 17.6 ± 7.2 (range: 10–29), indicating moderate to severe cognitive impairment (Table 1). All 10 patients were taking some medications that could increase the risk of falls, e.g., somnifacients, tranquilizers, or agents for benign prostatic hyperplasia, on the day of or the day before the fall (Table 2).

**Table 1:** Demographic characteristics of patients who experienced falls.

	Age	Sex	Disease	Vector	The types of paralysis	History	Rehabilitation	FAC	MMSE
Patient 1	82	Male	ICH	Trauma	Hemiplegia	DM	PT, OT, FES	2	–
Patient 2	70	Male	SDH	Trauma	Quadriplegia	–	PT, OT, CT, GT	3	26
Patient 3	49	Male	ICH	Spontaneous	Quadriplegia	DM	PT, OT, GT, FES	1	16
Patient 4	63	Female	SDH	Trauma	Quadriplegia	–	PT, OT, GT, FES	2	10
Patient 5	62	Male	SDH	Unknown	Quadriplegia	HTN	PT, OT, GT, FES	3	11
Patient 6	39	Female	SAH, ICH	Trauma	Quadriplegia	–	PT, OT, GT	4	29
Patient 7	53	Female	SDH	Spontaneous	Quadriplegia	DM	PT, OT, CT, GT, FES	1	16
Patient 8	62	Male	Tumor	Spontaneous	Hemiplegia	–	PT, OT, CT, GT, FES	4	–
Patient 9	68	Female	ICH, infarction	Spontaneous	Hemiplegia	HTN	PT, OT, CT, GT, FES	2	15
Patient 10	65	Male	ICH	Spontaneous	Hemiplegia	HTN	PT, OT, GT, FES	2	–

FAC, functional ambulation category; MMSE, mini-mental state examination; ICH, intracerebral hemorrhage; DM, diabetes mellitus; PT, physical therapy; OT, occupational therapy; FES, functional electrical stimulation; SDH, subdural hemorrhage; CT, cognition therapy; GT, gait training; HTN, hypertension; SAH, subarachnoid hemorrhage.

**Table 2:** Types and dosages of medications administered on the day before and the day of the fall.

	Patient 1	Patient 2	Patient 3	Patient 4	Patient 5	Patient 6	Patient 7	Patient 8	Patient 9	Patient 10
Day before the fall										
Somnifacient										
Circadin PR							2 mg*1			2 mg*1
Noiromin	300 mg*3	300 mg*3	300 mg*2	300 mg*3	300 mg*3					
Tranquilizer										
Buspirone HCl						15 mg*3				
Quetapin		25 mg*1	25 mg*1							
Razepam		1 mg*1		1 mg*1						
Rivotril			0.5 mg*1		0.5 mg*1	0.5 mg*4	0.25 mg*1			
Agent for benign prostatic hyperplasia										
Harnal-D									0.2 mg*1	
Proscar										5 mg*1
Tams	0.4 mg*1							0.4 mg*1		
Xatral XL										10 mg*1
Day of the fall										
Somnifacient										
Circadin PR							2 mg*1			2 mg*1
Noiromin	300 mg*3	300 mg*3	300 mg*2	300 mg*3	300 mg*3					
Tranquilizer										
Buspirone HCl						15 mg*3				
Quetapin		25 mg*1	25 mg*1							
Razepam		1 mg*1		1 mg*1						
Rivotril			0.5 mg*1		0.5 mg*1	0.5 mg*4	0.25 mg*1			
Agent for benign prostatic hyperplasia										
Harnal-D									0.2 mg*1	
Proscar										5 mg*1
Tams	0.4 mg*1							0.4 mg*1		
Xatral XL										10 mg*1

The 10 patients who experienced falls were classified as having a high risk of falls at the time of admission. Four of these patients (40%) fell during the day, while six (60%) fell at night. In five cases, a caregiver was present during the fall. On the other hand, no caregiver was present in the other five cases. Seven patients (70%) fell while getting out of bed, two patients (20%) fell while walking, and one patient (10%) fell during transfer from a wheelchair. Among the seven falls that occurred while getting out of bed, bed rails were raised in three cases and lowered in two cases. The status of the rails was unknown in two cases. Moreover, five out of these seven falls (71.4%) occurred at night.

Regarding the consciousness level during the fall, eight patients (80%) were alert, one patient was drowsy, and one patient was in a state of confusion. After the fall, seven patients (70%) did not report any injuries, while three patients (30%) had minor injuries with no abnormalities on imaging studies (Table 3).

**Table 3:** Fall-related characteristics.

	Time at fall	Presence of a caregiver at the time of the fall	Location	Presence of side rail	Consciousness state at the time of the fall	Presence of injury after the fall
Patient 1	Night	N	Bed	N	Drowsy	N
Patient 2	Night	Y	Bed	Y	Alert	Mild
Patient 3	Night	Y	Bed	Y	Alert	N
Patient 4	Day	N	Bed	Unknown	Confusion	Mild
Patient 5	Day	N	Bed	N	Alert	N
Patient 6	Night	Y	Bed	Y	Alert	N
Patient 7	Day	N	Walking	–	Alert	N
Patient 8	Night	Y	Walking	–	Alert	N
Patient 9	Day	N	Wheelchair	–	Alert	N
Patient 10	Night	Y	Bed	Unknown	Alert	Mild

N, no; Y, yes.

## 4 Discussion

In this study, among the 316 patients admitted during the study period, 10 patients (3.2%) experienced falls. This figure was lower than those reported in previous studies on fall rates in rehabilitation wards. For instance, Suzuki et al. (17) reported that 121 out of 256 patients (47.3%) admitted to a rehabilitation ward over a 21-month period experienced at least one fall, while Lee and Stokic (18) reported that 140 out of 1,472 patients (9.5%) admitted to a tertiary medical rehabilitation center over an 18-month period experienced at least one fall. Campanini et al. (19) documented falls in 11 out of 147 patients (7.5%) admitted to orthopedic, pulmonary, and neurological rehabilitation wards over a 6-month period. Several factors may have contributed to these discrepancies in study findings. The study periods ranged from 6 to 18 months; however, because the annual fall incidence rates were not standardized, direct comparisons between studies were limited. Moreover, Suzuki et al. (17), who reported a high fall rate, studied patients with a mean age of 68.6 ± 11.5 years—over 7 years older than the mean age of 61.3 ± 11.9 years in the present study. Given that age is a well-established risk factor that significantly influences fall incidence (20), differences in the age distribution of study participants may have contributed to the observed variations in results. Furthermore, fall incidence in rehabilitation wards was determined based on incident reports documented by medical staff, and variations in the institutional definitions of falls may have led to underreporting of similar events across studies. It is also important to consider that falls may have been underreported in cases where clinical supervision was not in place at the time of the incident or when patients or caregivers failed to report the fall. Consequently, the actual number of falls may have been higher than what was recorded in clinical reports.

The objective of this study was to investigate fall incidents that occurred in a single brain habilitation center over the past 3 years and to identify risk factors associated with falls. Additionally, this study aimed to provide practical solutions for fall prevention and suggest future research directions to support clinicians in developing effective preventive strategies. According to the findings of this study, multiple factors contributing to falls were identified.

Falls predominantly occurred during mobility, with seven out of 10 patients (70%) falling while attempting to get out of bed. Notably, five (71.4%) of these falls occurred at night, while two (28.6%) occurred during the day. This finding aligns with the finding of a previous study, in which over 60% of falls among patients with acquired brain injury occurred at night (21). The increased frequency of falls at night suggests that physical factors, such as impaired vision or medication-induced sleep disorders; environmental factors, such as dark lighting or a reduction in nursing staff during nighttime hours; and behavioral factors, such as the tendency of patients to move independently, can heighten fall risk. Visual impairment and sleep disorders are common symptoms in patients with acquired brain injury. In particular, 12 to 84% of patients with stroke experience visual deficits, such as visual loss or blurred vision, and over 50% experience sleep disturbances (22–24). Impaired vision can complicate the identification of surrounding obstacles and disrupt distance perception. Difficulties in clearly perceiving the environment and surrounding areas can increase the risk of falls (25). Moreover, in patients with stroke, sleep disorders often arise because of anxiety or depression, leading to the use of somnifacients or tranquilizers (22). These medications depress the central nervous system and induce sedation, which may result in muscle relaxation, impaired balance, or confusion (26). In particular, when patients who take somnifacient get up at night, they may experience increased confusion in the dark, increasing the risk of falls when getting out of bed or navigating to the bathroom. Furthermore, the residual effects of somnifacient can slow reaction times and reduce attention, increasing the risk of tripping during movement (27). Tranquilizers act on the central nervous system to suppress anxiety, tension, and agitation. However, they may also lead to muscle relaxation and impaired motor coordination (28, 29). Consequently, tranquilizers may diminish a patient’s ability to recognize and correct their body posture when feeling unsteady, making them more susceptible to falls (30, 31). These clinical findings support our hypothesis. In particular, among the 10 patients included in our study, nine had experienced a stroke. The five patients who fell around their beds at night were also stroke patients. All stroke patients were taking somnifacients and tranquilizers, which may have increased their risk of falls. In addition to physical factors, environmental or behavioral factors can play a significant role in increasing a patient’s risk of falls. Typically, hospital wards turn off all lights at night, creating a dark environment. However, at Yeungnam University Hospital—where this study was conducted—the lights in the ward hallway remain on at night to facilitate the movement of patients and medical staff. In contrast, hospital rooms are usually kept completely dark, with doors closed during sleeping hours. Additionally, patients frequently draw the curtains while sleeping, further diminishing visibility. Under such low-light conditions, patients may struggle to maintain adequate peripheral vision, making it difficult to locate and grasp safety handrails or determine where to place their feet (32). Moreover, staffing levels can influence patient safety. At Yeungnam University Hospital’s rehabilitation ward, the ratio of nursing staff to patients increases from 1 nurse per 8 patients during the day (6:30 a.m. to 9:30 p.m.) to 1 nurse per 13.3 patients at night (9:30 p.m. to 6:30 a.m.). This reduction in staffing may exacerbate safety risks, particularly in preventing falls during the night. Under these circumstances, more frequent monitoring of patient conditions becomes necessary during nighttime hours; however, the increased workload may lead to fatigue and stress among staff, potentially compromising the overall quality of patient care. Thus, optimizing staffing during critical hours, along with targeted environmental modifications, could be essential in reducing fall risks. Furthermore, in previous studies, patients who experienced falls were found to often refrain from seeking assistance before the fall because of reluctance to disturb family members or healthcare staff (5, 32). Moreover, patients undergoing rehabilitation after acquired brain injury may overestimate their physical capabilities and attempt movement without seeking help (33). To address this issue, hospitals can improve the environment by installing sensor lights to ensure safe navigation during sudden movements at night. Continuous education is also essential to encourage patients to refrain from wandering alone and to seek assistance when moving, thereby enhancing their safety.

Another noteworthy observation is that falls occurred despite the presence of caregivers and the presence of bed rails as environmental precautions. Among the 10 cases of falls, five cases (50%) occurred in the presence of caregivers. Regarding falls occurring around the bed, three out of seven cases (42.9%) occurred even with bed rails. These results suggest that falls could have occurred when the caregiver’s attention was diverted, the assistance provided was insufficient, the caregiver was unable to offer help, or the patient attempted to climb over the bed rails (32). Caregivers must pay close attention to patients with acquired brain injury, who have a high risk of falls. However, realistic limitations exist. For instance, the caregiver may occasionally step away without handing the patient over to medical staff or the caregiver may sleep at night, making continuous vigilance difficult. Moreover, if a patient falls toward the caregiver, the caregiver may be unable to counter the force. Consequently, both the patient and caregiver may collapse. To avoid these situations, it is crucial to conduct regular fall prevention training for caregivers to help them recognize and respond quickly to risky situations (34, 35). Furthermore, the introduction of monitoring equipment that enables real-time observation of a patient’s movements can facilitate immediate responses even if the caregiver’s attention is diverted (36, 37). The occurrence of falls even in the presence of bed rails suggests that such assistive devices alone may not ensure fall prevention. Several studies have revealed that bed rails do not guarantee patient safety (38–40). While bed rails can serve as a physical barrier against falls, attempts by patients to climb over or lean on them may pose a greater risk. Therefore, rather than simply installing assistive devices, it is vital to educate both patients and caregivers on the correct usage of these devices and to implement additional measures tailored to each patient’s physical condition. For instance, adjusting the height of beds for patients having a high risk of falls can create a safer environment when patients attempt to rise. Furthermore, placing frequently used items within easy reach of patients with mobility limitations can minimize unnecessary movements.

Finally, the fact that falls frequently occurred even among alert patients (80%) indicates that falls are not solely attributable to confusion or drowsiness. This suggests that even when a patient’s consciousness is intact, falls can occur due to physical limitations, impaired balance, misjudgment, or the side effects of medications. Among the 10 patients included in this study, eight patients (80%) had difficulty walking independently (FAC score < 4), four patients (40%) had hemiplegia, and six patients (60%) had quadriplegia. Furthermore, five patients (50%) exhibited impaired cognitive function (MMSE score < 24), and four patients (40%) were taking medications, such as those for benign prostatic hyperplasia, in addition to somnifacients and tranquilizers. Motor dysfunctions make it challenging to maintain stability or balance during processes like getting in or out of bed or during movement. Damage to neural pathways following acquired brain injury often reduces muscle strength in certain body parts (41). In particular, weakened muscles in the legs or torso can make it difficult to adequately support body weight when rising from a bed, leading to a higher risk of imbalance (42, 43). Insufficient muscle strength can impair the ability to stabilize the body during weight-bearing activities, such as standing or moving, thereby increasing the risk of falls (44). Moreover, when paralysis occurs as a result of acquired brain injury, patients can face even greater difficulties in maintaining body balance (45). For example, when rising from a bed, if one leg cannot adequately support weight or if balance cannot be maintained with one arm, the risk of falling is heightened. Severe muscle weakness or paralysis can also lead to asymmetric gait patterns, complicating normal walking (46). Furthermore, a decline in motor coordination, which facilitates smooth and accurate movements through the simultaneous control of multiple muscles, may result in uncoordinated and inaccurate movements (47). Excessive or diminished muscle tone following acquired brain injury can further degrade balance and stability, increasing the risk of falls (48). Therefore, to reduce the risk of falls, it may be beneficial for rehabilitation therapies to emphasize tailored interventions that specifically address fall prevention (49). Strength training could target the most affected muscle groups, such as lower limb and core muscles, to enhance postural control and improve weight-bearing capacity. Balance training might be optimized by incorporating exercises such as marching in place, single-leg standing, and weight-shifting movements, which could help improve stability during daily activities (50). Additionally, functional electrical stimulation might be considered to help activate weakened muscles, thereby supporting both mobility and stability (51). Furthermore, educating patients on the proper use of assistive devices, such as walkers, canes, or crutches, can help maintain balance and enhance stability during movement (48). Cognitive decline following acquired brain injury can also increase the risk of falls. Cognitive impairment is recognized as an independent risk factor for falls. The risk of falling is approximately 2.7 times higher in individuals with cognitive impairment than in those without it (52). Impaired cognitive function slows down behavior and reaction times, leading to delayed responses in unexpected situations and a resultant higher risk of falls (53). Furthermore, cognitive decline can reduce spatial awareness and attention, making it challenging to accurately assess the surrounding environment (54). This impairment affects critical abilities needed to maintain balance or avoid obstacles, thereby increasing the risk of falls. To alleviate the risk of falls associated with cognitive decline, various therapeutic approaches can be employed. Cognitive training programs can enhance attention, judgment, and spatial awareness, helping patients to safely navigate and respond to hazardous situations (55). Moreover, occupational therapy can reduce errors and inattention related to cognitive impairment by repeatedly practicing daily living activities (27). Psychological support and education can also play a significant role in fall prevention by alleviating patients’ fears of falling and enhancing their awareness of fall risks (56). Medications are a modifiable risk factor that can increase the risk of falls. The agents for benign prostatic hyperplasia used by the participants in this study included alpha-blockers, which relax the muscles of the prostate and bladder neck, and 5-alpha reductase inhibitors, which reduce the size of the prostate. These drugs can alleviate urinary difficulties caused by prostate enlargement; however, they can also affect blood vessels and lower blood pressure (57, 58). This action can lead to orthostatic hypotension, increasing the risk of sudden dizziness and falls on standing up (59). Alpha-blockers can also reduce muscle tone by blocking alpha-1 receptors, potentially decreasing stability and making it harder to maintain balance during walking or other daily movements (60). Therefore, when prescribing medications to patients having a high risk of falls following acquired brain injury, it is preferable to avoid drugs that can further increase the risk of falls. However, if the use of such medications is unavoidable, they should be administered at the lowest effective dose for the shortest duration possible (61). Moreover, providing thorough education to patients and caregivers about the potential side effects of these medications is crucial to help mitigate the risk of falls.

This study has several limitations. First, we did not identify which factors are independent risk factors for falls by comparing patients who experienced falls with those who did not. Second, the analysis was based on a small sample from a single institution. Therefore, future research should combine data from multiple institutions to establish and analyze a large-scale database of falls. This would provide a more comprehensive understanding of the risk factors for falls and contribute to the development of more effective fall prevention and management strategies.

## 5 Conclusion

This study investigated fall incidents that occurred in a single brain rehabilitation center and identified the associated risk factors and prevention strategies. The results indicated that falls primarily occurred during patient mobility from the bed, particularly at night. Moreover, frequent falls occurred even when patients were in a state of clear consciousness. This suggests that physical factors, such as visual impairments and side effects of medication; environmental factors, such as dark lighting or insufficient nursing staff coverage; and behavioral factors, such as patients’ attempts to move without assistance, could contribute to falls. Falls even occurred despite the presence of caregivers and bed rails, highlighting the practical limitations of caregiver and assistive device roles. In conclusion, a multifaceted approach that considers physical, environmental, and behavioral factors is necessary for fall prevention. The implementation of various measures—including nighttime lighting, comprehensive training on the use of assistive devices, ongoing education for caregivers, the integration of monitoring equipment, patient-specific rehabilitation programs, and meticulous management of medications that may increase fall risk—could prove effective in reducing falls. These strategies may help create a safer environment and support fall prevention efforts. Additionally, future research could focus on identifying independent risk factors for falls by employing a multivariate approach that compares patients who experienced falls with those who did not. Moreover, expanding studies to multiple institutions with larger sample sizes could enhance the generalizability of findings and provide a more comprehensive understanding of fall risk factors across diverse healthcare settings. Furthermore, exploring emerging technologies, such as real-time monitoring systems and artificial intelligence-based fall prediction models, holds the potential to offer innovative solutions for proactive fall prevention in rehabilitation environments. By addressing these areas, future research may contribute to more effective and data-driven fall prevention strategies, ultimately supporting improvements in patient safety and rehabilitation outcomes.

## Data Availability

The original contributions presented in the study are included in the article/supplementary material, further inquiries can be directed to the corresponding author.
